# Fluoroquinolone-resistant *Salmonella* Paratyphi A

**DOI:** 10.3201/eid1101.040145

**Published:** 2005-01

**Authors:** Takuya Adachi, Hiroko Sagara, Kenji Hirose, Haruo Watanabe

**Affiliations:** * Yokohama Municipal Citizen’s Hospital, Yokohama, Japan; † National Institute of Infectious Diseases, Tokyo, Japan

**Keywords:** letter, Salmonella paratyphi A, carrier state, drug resistance, fluoroquinolones, point mutation, DNA gyrase, DNA topoisomerase IV

**To the Editor**: Fluoroquinolones have been the drug of choice for treating typhoid and paratyphoid fever since the beginning of the 1990s. Multidrug-resistant strains began to prevail in disease-endemic areas, and former first-line antimicrobial drugs, such as chloramphenicol, were sometimes ineffective (*1*). In recent years, however, strains with decreased susceptibility to quinolones have emerged, and clinical treatment failure is a serious concern (*2*–*5*).

An 87-year-old woman was referred from a local clinic to Yokohama Municipal Citizen’s Hospital in July 2002 because *Salmonella enterica* serovar Paratyphi A was detected in her urine. She had no subjective symptoms such as pain on urination or urinary urgency, and her temperature was normal. She had never had paratyphoid fever, and she had not traveled abroad. No other person in the community had paratyphoid. Before being admitted to the hospital, she had experienced frequent episodes of urinary tract infection and had been empirically treated each time with oral antimicrobial drugs, including ciprofloxacin. She had been given a dose of 600 mg/day for 7 days, 25 times in the last 4 years.

The patient did not display any abnormal findings on physical examination. *S*. Paratyphi A was not detected in the urine but was confirmed in the stool; therefore, the previous report of bacteriuria could have been due to contamination of a urine sample with feces. An ultrasound showed a polyp and multiple stones in her gallbladder. A carrier state was suspected. Bile was obtained by duodenal aspiration and was positive for *S*. Paratyphi A. The patient was considered to be an asymptomatic cholecystic carrier of *S*. Paratyphi A.

On disk diffusion susceptibility testing, the isolate was resistant to nalidixic acid (NA) and to ofloxacin. The MIC of ofloxacin was as high as 256 µg/mL, and the MIC of ciprofloxacin was128 µg/mL (Table). An open cholecystectomy was performed for treatment of the polyp, the stones, and the highly quinolone-resistant bacteria. A routine perioperative intravenous antimicrobial agent, cefmetazole, was administered as surgical prophylaxis. The polyp was malignant, and the operation was curative. The patient resumed normal activities, and had no further fecal excretion of *S*. Paratyphi A.

**Table:** MICs of antimicrobial agents for the isolate of *Salmonella enterica* serovar Paratyphi A*

	MIC (µg/mL)	
Antimicrobial agent	Etest	Broth microdilution
Ampicillin	4	ND
Chloramphenicol	16	ND
Gentamicin	0.06	ND
Kanamycin	1	ND
Streptomycin	0.75	ND
Sulfamethoxazole/trimethoprim	0.5	ND
Tetracycline	8	ND
Cefoperazon	1.5	ND
Cefotaxime	0.38	ND
Ceftriaxone	0.19	ND
Imipenem	0.19	ND
Aztreonam	0.125	ND
Fosfomycin	64	ND
Nalidixic acid	>256	ND
Norfloxacin	>256	1024
Ofloxacin	>32	256
Sparfloxacin	>32	256
Ciprofloxacin	>32	128
Levofloxacin	ND	128
Tosufloxacin	ND	128

*ND, no data.

Polymerase chain reaction (PCR) amplification and DNA sequencing were conducted to detect mutations responsible for the fluoroquinolone resistance. Nucleotide sequences of *gyrA*, *gyrB*, *parC*, and *parE* genes were investigated. The primers used for PCR amplification and DNA sequencing have been previously described (*6*,*7*). An ABI Prism dye terminator cycle sequencing kit (Perkin-Elmer, Applied Biosystems, Foster City, CA) and an automated sequencer (311A; Perkin-Elmer, Applied Biosystems) were used. The isolated strain possessed triple point mutations. The first 2 mutations were in the *gyrA* gene, which encodes DNA gyrase, at codon 83 (TCC to TTC), which substitutes phenylalanine for serine, and at codon 87 (GAC to AAC), which substitutes asparagine for aspartic acid. The third mutation was in the *parC* gene, which encodes DNA topoisomerase IV, at codon 84 (GAA to AAA), which substitutes lysine for glutamic acid. No mutations were found in *gyrB* and *pare E*.

*S*. Typhi and Paratyphi A with decreased susceptibility to fluoroquinolones emerged on the Indian subcontinent, Southeast Asia, and Central Asia in the mid-1990s (*2*–*5*). On disk diffusion testing, these strains were NA-resistant, and susceptible to ofloxacin or ciprofloxacin; however, the MICs of ciprofloxacin increased to 0.25–4 μg/mL, 10- to 100-fold higher than the usual NA–susceptible strains (*5*,*8*,*9*). NA-resistant strains of *S*. Typhi have 1 point mutation at the target site of quinolones, DNA gyrase, in the quinolone resistance–determining region of *gyrA*, either at codon 83 or codon 87 (*2*,*3*). Several epidemics of typhoid and paratyphoid fever caused by NA-resistant strains with clinical failure of quinolone treatment have been reported (*4*,*5*).

An experimental attempt had been previously made to induce the production of strains with high-level fluoroquinolone resistance by culturing strains of *S*. Typhi and Paratyphi A in ciprofloxacin-supplemented medium (*7*). One of these in vitro–induced, high-level resistant strains of *S*. Paratyphi A had triple mutations in the *gyrA* gene at codons 83 and 87 and in the *parC* gene at codon 84, which are the same triple mutations as those seen in the current in vivo case.

Full fluoroquinolone resistance has already emerged in the community in nontyphoid *Salmonella* species. In a clinical isolate of *S. enterica* serovar Typhimurium, the MIC of ciprofloxacin was 32 µg/mL, and mutations in both *gyrA* and *gyrB* were noted (*10*).

Our findings strongly suggest that high-level quinolone resistance was induced through the long-term carrier state of *S*. Paratyphi A under selective pressure of frequent quinolone administration. The resistance is associated with the 2 mutation sites in *gyrA* and 1 site in *parC*, and multiple point mutations are likely related to the acquisition of full resistance. Physicians should be aware of the emergence of *S*. Typhi and Paratyphi A, as well as nontyphoid *Salmonella* species, which are highly quinolone-resistant.
